# Gut microbiome with *RAS* mutation and chemotherapy response in patients with advanced or metastatic colorectal cancer: a pilot, exploratory study

**DOI:** 10.3389/fonc.2025.1565661

**Published:** 2025-09-01

**Authors:** Jwa Hoon Kim, Boyeon Kim, Jiwon Lee, Jin Kim, Jung-Myun Kwak, Soohyeon Lee

**Affiliations:** ^1^ Division of Medical Oncology, Department of Internal Medicine, Korea University College of Medicine, Seoul, Republic of Korea; ^2^ Cancer Research Institute, Korea University College of Medicine, Seoul, Republic of Korea; ^3^ Department of Surgery, Korea University College of Medicine, Seoul, Republic of Korea

**Keywords:** colorectal cancer, gut microbiome, RAS mutation, chemotherapy response, prognosis

## Abstract

The gut microbiome plays a pivotal role in tumor–microenvironment interactions, inflammation modulation, and immune regulation, thereby affecting the response to anticancer therapy. This pilot study investigated gut composition according to clinical characteristics and its association with chemotherapy response in patients with metastatic colorectal cancer (mCRC). Seventeen patients were treated with first-line chemotherapy at Korea University Anam Hospital between 2021 and 2023. Stool samples were collected from 15 patients at baseline, during chemotherapy, or at the time of disease progression, and 16S rRNA sequencing was performed. As a result, among lifestyle factors affecting the development of CRC, smoking habits showed weak differences in beta diversity. Non-smokers predominantly harbored bacteria such as *Butyricicoccaceae*, *Ruminococcaceae*, *Faecalibacterium*, and *Lachnospiraceae*_*NK4A136*_*group*, whereas Smokers were associated with *Actinomyces* and *Solobacterium*. In terms of baseline characteristics and chemotherapy response, beta diversity exhibited notable differences according to *RAS* mutation status, and LEfSe analysis indicated that *Holdemanella*, *Anaerostipes*, and *Collinsella* were significantly enriched in patients with *RAS* mutations. Chemotherapy Responders harbored more beneficial bacteria, notably *Lactobacillus*, despite the lack of differences in diversity between the responder and Non-responder groups. According to disease control during follow-up, *Bifidobacterium* abundance significantly increased in the non-progressive disease group. This study suggests that gut microbiome composition is associated with smoking history, RAS mutation status, and chemotherapy response in patients with mCRC. These findings highlight the potential role of the gut microbiome as a biomarker to predict treatment response and prognosis, with its composition shaped by both host lifestyle factors and genetic mutations.

## 1 Introduction

Colorectal cancer (CRC) develops through the accumulation of mutations in colonic epithelial cells, which promote the transition from normal mucosa to adenocarcinoma (1). *APC* is a tumor suppressor gene that regulates cell adhesion and migration, maintenance of genome stability, and apoptosis (1, 2). It is known to be the gatekeeper gene during the adenoma–carcinoma sequence of CRC. In addition, several driver mutations, such as *KRAS*, *BRAF*, *PIK3CA*, *SMAD4*, and *TP53*, are associated with the development and progression of CRC (2). Several environmental factors contribute to the occurrence and accumulation of these somatic mutations. Numerous carcinogens, including fat, red and processed meat, alcohol, chemicals, and smoking, pass through the gastrointestinal (GI) tract and contribute to the development and progression of cancer, including CRC (3, 4). The increasing incidence of CRC in historically low-risk areas, such as Eastern Asia, is attributed to the so-called Western lifestyle (1, 5, 6).

The gut microbiome comprises a diverse group of microorganisms inhabiting the gastrointestinal (GI) tract that can be altered by daily lifestyle and dietary patterns (3, 7, 8). However, these habits cannot be determined using only one simple marker. Instead, the gut microbiome passing through the GI tract, which reflects the overall environment of the GI tract, can serve as a surrogate indicator. It plays a critical role in digestion, immunity, and overall health, and may influence cancer risk and progression (3, 7, 9). Certain bacterial species—including *Fusobacterium nucleatum*, *Bacteroides fragilis*, and *Escherichia coli*—are more abundant in CRC tissues than in normal tissues, indicating their potential role in cancer development (10). In contrast, beneficial bacteria, such as certain strains of *Bifidobacterium and Lactobacillus*, have shown anti-inflammatory and anti-carcinogenic effects in the colon (11).

Moreover, increasing evidence suggests that the gut microbiome is associated with cancer prognosis and response to chemotherapy. Specifically, *Faecalibacterium* and *Ruminococcaceae* were frequently observed in patients who responded well to cancer treatment (Responders), whereas *Bacteroides* was observed more frequently in Non-responders. Novel strategies for modulating the gut microbiome have been attempted in cancer treatment.

We aimed to investigate the characteristics of the gut microbiome in relation to clinical features and to explore its association with chemotherapy response in patients with locally advanced or metastatic CRC (mCRC).

## 2 Methods

We conducted a prospective pilot exploratory study to examine the diversity and composition of the gut microbiome according to clinicogenomic factors and chemotherapy responses in patients with mCRC. Written informed consent was obtained from all patients. This study was approved by our Institutional Review Board (approval number: 2021AN0403). The trial procedures were performed in accordance with the Declaration of Helsinki and the Guidelines for Good Clinical Practice.

### 2.1 Patients and data collection

This study enrolled patients with mCRC who were treated with first-line systemic chemotherapy between October 2021 and February 2023 at Korea University Anam Hospital. Eligible patients were those aged ≥19 years, with histologically or cytologically confirmed locally advanced or metastatic adenocarcinoma of the colon or rectum, Eastern Cooperative Oncology Group (ECOG) scores of 0–2, and no prior history of palliative systemic chemotherapy or antibiotic use.

The baseline clinical data included age; sex; smoking history; alcohol history; *KRAS, NRAS*, and *BRAF* mutation status; microsatellite instability (MSI) status; and prior radiotherapy. Data on chemotherapy regimens and responses were collected. *RAS* (*KRAS* and *NRAS*) and *BRAF* mutations were identified using polymerase chain reaction (PCR) or next-generation sequencing. 5-fluorouracil, leucovorin, and oxaliplatin (FOLFOX) or 5-fluorouracil, leucovorin, and irinotecan (FOLFIRI), with or without biological agents targeting vascular endothelial growth factor (VEGF) or epidermal growth factor receptor (EGFR), was administered every 2 weeks until disease progression or unacceptable toxicity. Tumor response was assessed according to the Response Evaluation Criteria in Solid Tumors (RECIST) version 1.1 using computed tomography (CT) or magnetic resonance imaging (MRI) every 6 weeks during treatment. Short-course radiotherapy was administered to patients with rectal cancer.

### 2.2 Stool sample collection and 16S rRNA sequencing

Stool samples were self-collected by participants 1 day prior to their scheduled visit to Korea University Anam Hospital. Samples were obtained either before the initiation of systemic chemotherapy, during treatment, or at the time of disease progression. The OM-200 kit (DNA Genotek, Canada) was used according to the manufacturer’s instructions to collect stool samples. All stool samples were stored in the participant’s home freezer (−20°C), packed with ice packs, transported to the laboratory on ice, and stored in a deep freezer (−80°C), minimizing freeze–thaw cycles. After thawing, each sample was manually homogenized using a sterile tip, and small aliquots (0.2 g) were collected in a 2.0 mL microtube for microbiome and metabolome analyses. Fecal bacterial genomic DNA was extracted using the Mag-Bind^®^ Universal Pathogen Kit (Omega Bio-tek, Norcross, GA, US). 16S rRNA sequencing was performed to analyze the composition and diversity of the gut microbiome.

### 2.3 Statistical analysis

Alpha diversity was quantified as the number of observed amplicon sequence variants (ASVs) and Chao1, Shannon, and Simpson indices. Wilcoxon signed-rank tests were used to evaluate differences in diversity among samples. Bray–Curtis and weighted UniFrac distance matrices for beta diversity were obtained, and q values were calculated using QIIME 2. These matrices were then imported into R to generate principal coordinate analysis plots. The linear discriminant analysis (LDA) effect size (LEfSe) algorithm was applied to identify taxonomic biomarkers and analyze them at the family and genus levels. Default parameters were used for significance (p < 0.05) and the LDA threshold (LDA score > 2.0).

Progression-free survival (PFS) was calculated from the date of initiation of first-line chemotherapy to the date of progression or death from any cause. Overall survival (OS) was calculated from the date of initiation of first-line chemotherapy to the date of death from any cause. Survival rates were estimated using the Kaplan–Meier method, and the log-rank test was used to compare differences between curves.

All analyses were performed using R packages (Qiime2R (version 0.99.6), Microbial (version 0.0.20), Microbiomeutilities (version 1.00.17)) and GraphPad Prism (version 9.0). Differences in the abundance of each microbial species were determined using the Mann–Whitney test, and p values < 0.05 were considered statistically significant.

## 3 Results

### 3.1 Patient characteristics

A total of 17 patients were included in this study. **Table 1** summarizes the baseline patient characteristics. The median age was 61 years (range, 35–73 years), and 47.1% of the patients were men. The cohort included patients with *RAS*-mutated CRC (n = 8, 47.1%) and *BRAF*-mutated CRC (n = 1, 5.9%). All patients had microsatellite stable CRC. In terms of chemotherapy response, there were nine responders (complete response and partial response) and eight Non-responders (stable disease and progressive disease). At a median follow-up of 15.9 months (95% confidence interval [CI], 13.49–18.27), the median PFS was 10.9 months (95% CI, 7.12–14.64), and the median OS was not reached because none of the patients had died at the time of the analysis.

**Table 1:** Patients’ baseline characteristics.

Baseline characteristics	N=17
Age (years)	(35–73)
Sex	
Male	8
Female	9
Status	
Locally advanced disease	1
Initially metastatic disease	11
Recurrent disease	5
Primary location	
Rectum	7
Colon	10
Gene mutations	
* RAS*	8^a^
* BRAF*	2
Deficient MMR or MSI high	0
Sites of metastasis	
Liver	10
Lung	7
Peritoneum	2
Bone	0
Brain	1
Prior radiotherapy	
Short-course radiotherapy to rectum	4
Radiotherapy to left obturator lymph node	1
Regimens of chemotherapy	
5-fluorouracil + oxaliplatin	2
5-fluorouracil + oxaliplatin + bevacizumab	9
5-fluorouracil + oxaliplatin + cetuximab	1
5-fluorouracil + oxaliplatin + anti-HER2 agent	1
5-fluorouracil + irinotecan + bevacizumab	1
5-fluorouracil + irinotecan + cetuximab	3

^a^One patient had *NRAS* mutation and *KRAS* wild-type.

Among the 17 patients, two failed to collect stool samples. Thirty stool samples were collected from 15 patients with mCRC: before chemotherapy (n = 15), during chemotherapy (n = 10), and at disease progression (n = 5). All samples met the QC requirements for 16S rRNA sequencing.

### 3.2 Comparing the gut microbiome by lifestyle factors: alcohol consumption and smoking habits

**Figure 1:**
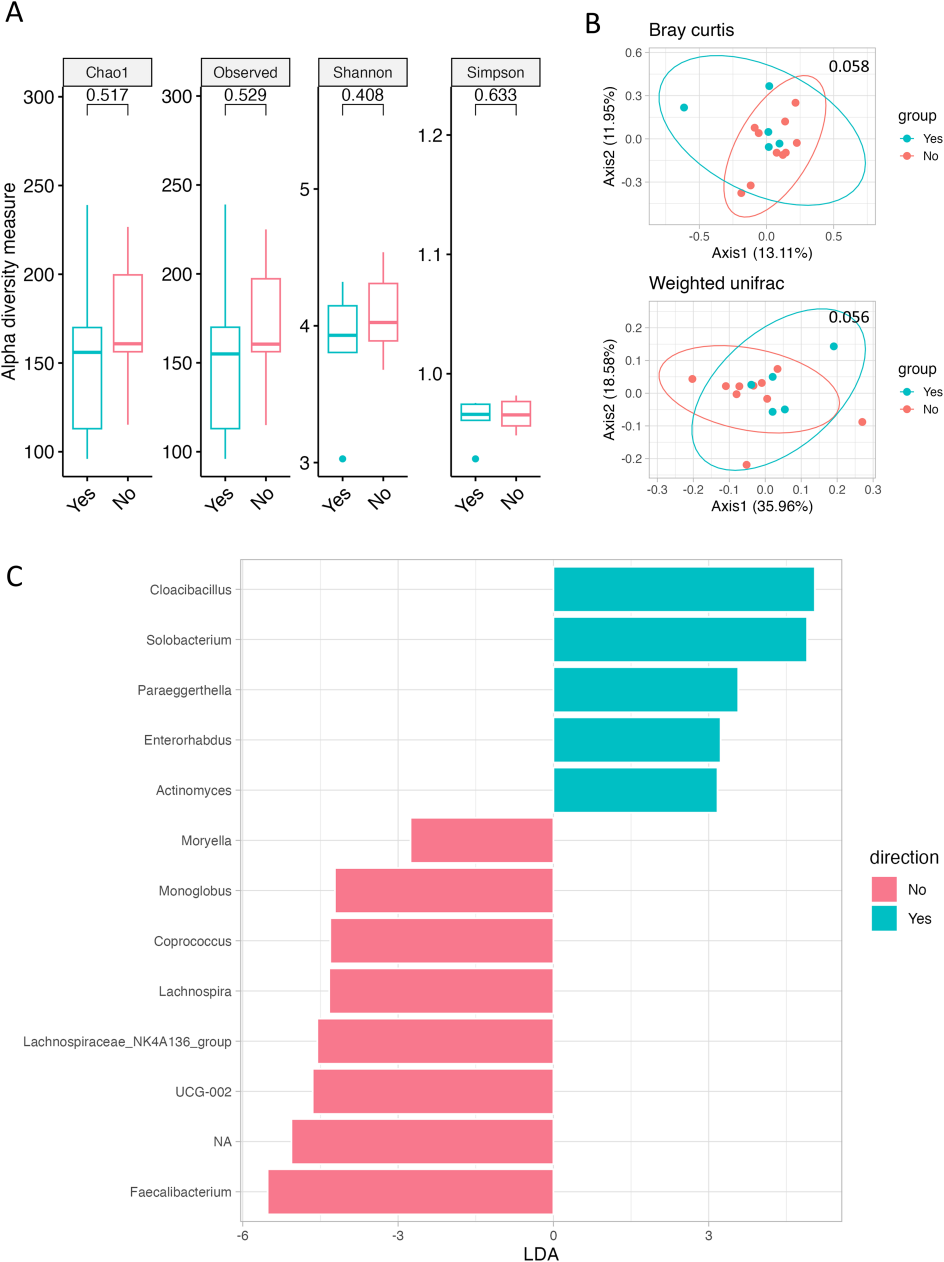
Gut microbiome according to smoking history. **(A)** Alpha diversity, as measured by the Chao1, Observed ASVs, Shannon, and Simpson indices, is depicted for both Smokers (green) and Never-smokers (red). **(B)** PCoA plots represent beta diversity, specifically Bray–Curtis (top) and weighted UniFrac (bottom). In these plots, each point represents a single sample, color-coded as Smokers (green) or Never-smokers (red). **(C)** Genus-level LEfSe analysis identified taxa with differential abundance in Smokers (green) and Never-smokers (red).

We compared the gut microbiome according to current alcohol intake and smoking status to assess its association with lifestyle habits known to potentially influence CRC development. No differences in alpha or beta diversity of the gut microbiome were found according to alcohol intake (**Supplementary Figures 1A, B**). Differences in alpha diversity according to smoking status were not significant; however, beta diversity indicated by Bray–Curtis and weighted UniFrac showed a weak difference (p = 0.058 and p = 0.056, respectively; **Figures 1A, B**). Furthermore, variations were observed in the microbial community based on smoking status. Non-smokers predominantly harbored bacteria such as *Butyricicoccaceae*, *Ruminococcaceae*, *Faecalibacterium*, and *Lachnospiraceae*_*NK4A136*_*group*, whereas Smokers mainly harbored *Actinomyces* and *Solobacterium* (**Figure 1C**, **Supplementary Figure 2**).

### 3.3 Analyzing gut microbial diversity and composition in relation to *RAS* mutation

**Figure 2:**
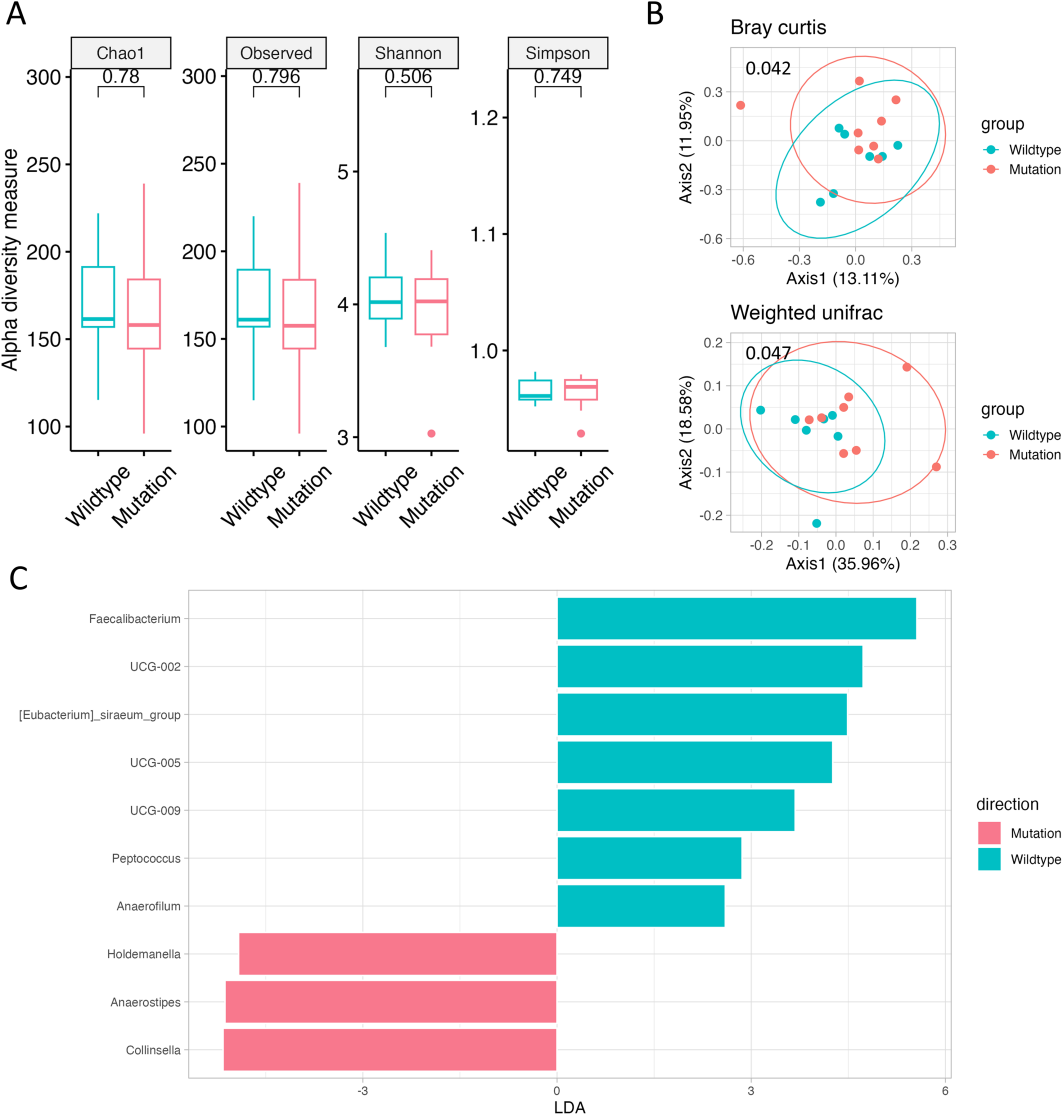
Gut microbiome according to *RAS* mutation. **(A)** Alpha diversity, as measured by the Chao1, Observed ASVs, Shannon, and Simpson indices, is depicted for both *RAS* Wild-type (green) and *RAS*-mutated (red) CRC. **(B)** Principal coordinate analysis (PCoA) plots represent beta diversity, specifically Bray–Curtis (top) and weighted UniFrac (bottom). In these plots, each point represents a single sample, color-coded as *RAS* Wild-type (green) or *RAS*-mutated (red) CRC. **(C)** Genus-level LEfSe analysis identified taxa with differential abundance in *RAS* Wild-type (green) and *RAS*-mutated (red) CRC.

We aimed to observe changes in the gut microbiome according to genetic mutations. However, due to the presence of BRAF mutations in only one patient and the absence of patients with deficient mismatch repair (MMR) or high MSI, statistical analysis for these factors was not feasible (data not shown). Regarding *RAS* mutations, although there were no significant changes in alpha diversity, beta diversity, as represented by Bray–Curtis and weighted UniFrac, exhibited notable differences (p = 0.042 and p = 0.047; **Figures 2A, B**). LEfSe analysis identified *Holdemanella*, *Anaerostipes*, and *Collinsella* as significantly enriched in patients with *RAS* mutations (**Figure 2C**, **Supplementary Figure 3A**). Additionally, at the family level, the frequency of *Coriobacteriaceae* increased in patients with *RAS* mutations (**Supplementary Figures 3B, C**).

### 3.4 Assessment of response to chemotherapy and baseline gut microbiome

**Figure 3:**
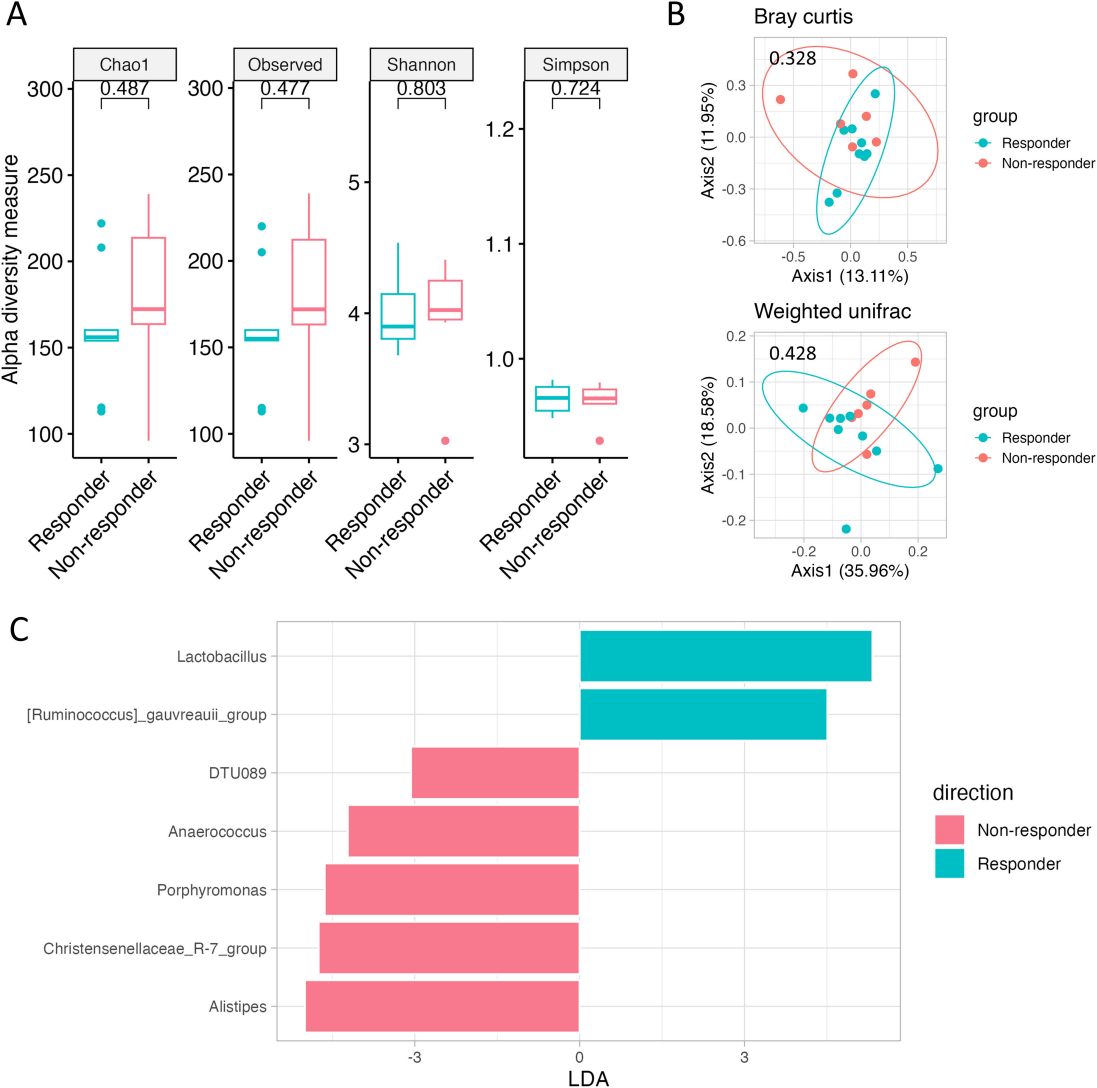
Gut microbiome according to chemotherapy response. **(A)** Alpha diversity, as measured by the Chao1, Observed ASVs, Shannon, and Simpson indices, is depicted for both Responders (green) and Non-responders (red). **(B)** PCoA plots represent beta diversity, specifically Bray–Curtis (top) and weighted UniFrac (bottom). In these plots, each point represents a single sample, color-coded as Responders (green) or Non-responders (red). **(C)** Genus-level LEfSe analysis identified taxa with differential abundance in Responders (green) and Non-responders (red).

After grouping the 15 patients into Responders (n = 9) and non-responders (n = 6) and analyzing the baseline gut microbiome before chemotherapy, no differences in alpha or beta diversity were found (**Figures 3A, B**). Nonetheless, the baseline samples of Responders contained numerous beneficial bacteria, including *Lactobacillus* spp. (**Figure 3C**, **Supplementary Figure 4**).

### 3.5 Exploring changes of microbial abundance according to disease control with chemotherapy

**Figure 4:**
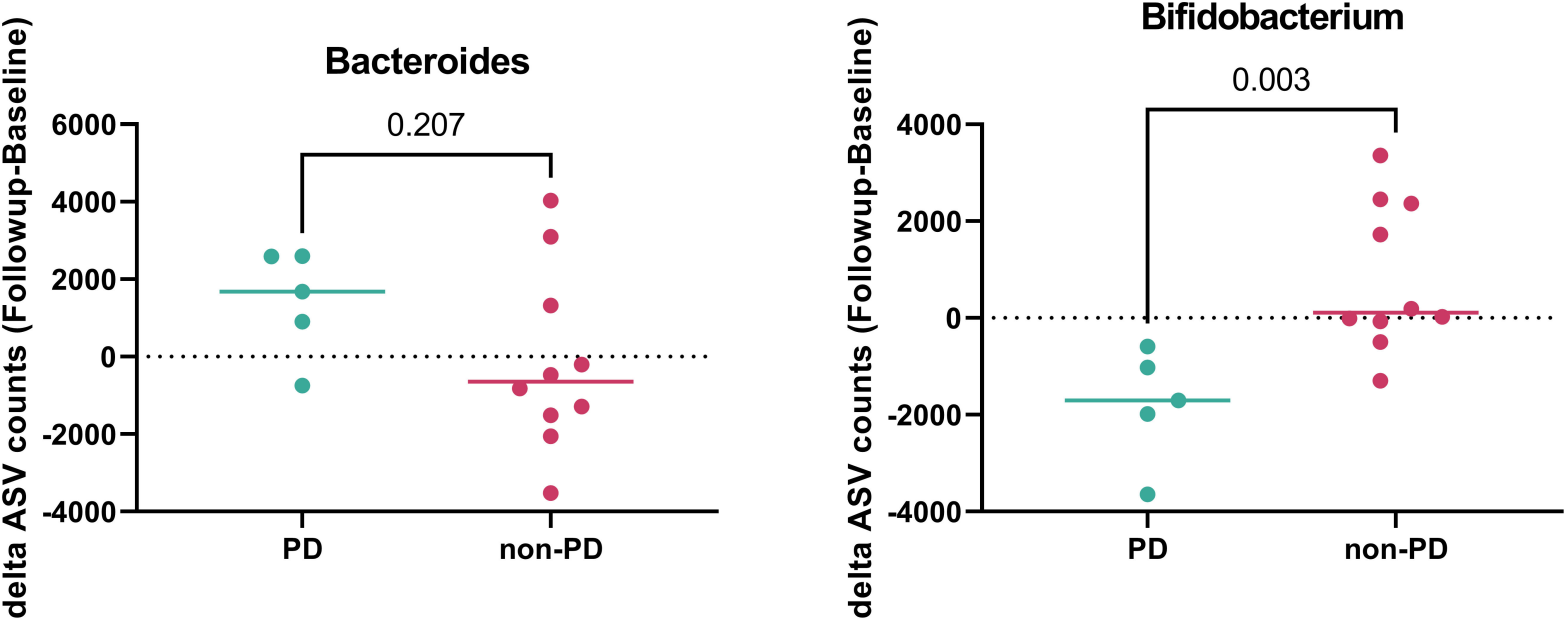
Gut microbiome according to disease control during follow-up. The abundance of *Bacteroides* and *Bifidobacterium* in patients with progressive disease (PD) (green) and non-PD (red).

Patients were grouped into progressive disease (PD) and non-PD groups based on whether their disease was controlled at the time of stool collection. We compared changes in the gut microbiome between baseline and follow-up. Among the investigated gut microbiota, there were no changes in the abundance of *Enterococcus*, *Faecalibacterium*, *Peptostreptococcus*, *Streptococcus*, or *Lactobacillus* between baseline and follow-up. Notably, *Bacteroides* tended to increase in the follow-up samples from the PD group (p = 0.2065), whereas *Bifidobacterium* significantly increased in the follow-up samples from the non-PD group (p = 0.0027; **Figure 4** and **Supplementary Figure 5**).

## 4 Discussion

This exploratory pilot study investigated the gut microbiome in relation to lifestyle factors, clinicopathologic characteristics, and chemotherapy responses in patients with mCRC. The findings suggest that smoking habits alter the composition of the gut microbiome. Furthermore, this study highlights a potential association between RAS mutation status and the gut microbiome, which may ultimately influence chemotherapy response in patients with mCRC.

The gut microbiome comprises a diverse community of microorganisms residing in the gastrointestinal tract, and its composition can be influenced by various lifestyle factors. Our research team previously conducted a separate study involving colorectal cancer patients after surgical resection, in which a high-fiber diet was associated with an increased abundance of beneficial bacteria such as *Prevotella*. Furthermore, patients who experienced improvement in diarrhea symptoms showed elevated levels of *Akkermansia* and short-chain fatty acids (SCFAs) (12). In the present study, we also observed differences in gut microbiome composition according to smoking habits. Smokers had a higher prevalence of *Actinomyces* and *Solobacterium*—considered harmful microbiome components—than non-smokers among patients with mCRC. The diversity of the gut microbiome may also be more disrupted in Smokers than in non-smokers, given that this study showed a weak difference. Although the mechanisms by which smoking affects CRC development are not yet clear, the gut microbiome may play a mediating role (13). No relationship was found between alcohol consumption and the gut microbiome in this study. Although the composition of the gut microbiome cannot be explained by simple factors and requires comprehensive consideration of all environmental factors, these findings suggest that lifestyle factors, including diet and smoking, may contribute to alterations in the gut microbiome, which in turn could potentially influence clinical outcomes and prognosis in patients with CRC.

Notably, there were differences in the abundance and composition of the gut microbiome according to *RAS* mutation status in this study. Host genetics may influence the gut microbiome. A genome-wide association study (GWAS) of host genetic variation in microbiome taxa identified 31 loci affecting the gut microbiome, and the lactase gene locus was associated with *Bifidobacterium* abundance (14). In contrast, the gut microbiome has been shown to cause DNA damage, providing a driver for somatic mutations in preclinical studies (15). Colibactin produced by *E. coli* containing a pks island can cause double-strand breaks in mammalian DNA, promoting genome instability and increasing the mutation rate (16, 17). *H. pylori* and enteropathogenic *E. coli* can disrupt mismatch repair (MMR), leading to the deletion of MMR proteins (18, 19). A previous study reported a relationship between *KRAS* mutations and the gut microbiome in CRC (20). *Roseburia*, *Parabacteroides*, *Metascardovia*, *Staphylococcus*, and *Bacillales* are associated with *KRAS* mutation (20). As a proof of principle, tumor-associated species such as *Fusobacterium* and *Bacteroides* are positively correlated with cancer-related inflammatory pathways and negatively associated with cellular adhesion machinery (21). However, clinical data regarding the association between the gut microbiome and cancer-associated somatic mutations are lacking.

Our study provides clinical evidence for potential interactions between the gut microbiome and *RAS* mutations in mCRC. *Anaerostipes*, *Collinsella*, and *Holdemanella* were significantly more abundant in *RAS*-mutated CRC than in *RAS* Wild-type CRC, whereas *Faecalibacterium* and *Eubacterium* were significantly more abundant in *RAS* Wild-type CRC than in *RAS*-mutated CRC. *Bacteroides*, which is also associated with the development and progression of CRC, was more prevalent in *RAS*-mutated CRC than in *RAS* Wild-type CRC, although the difference was not statistically significant. *Faecalibacterium*, *Eubacterium*, as well as *Anaerostipes*, *Collinsella*, *Holdemanella*, and *Bacteroides* are representative gut microorganisms involved in CRC development and progression (1, 7, 11). In particular, *Faecalibacterium* is associated with a better treatment response, whereas *Anaerostipes* and *Bacteroides* are associated with poor treatment response (22). This may be one of the reasons for the poor prognosis of *RAS*-mutated CRC compared with *RAS* Wild-type CRC. Although preclinical functional studies are essential to elucidate the role of specific gut microbiota in prognosis, our study raises the possibility that differences in the gut microbiome between *RAS*-mutated and wild-type CRC may explain the different prognoses of CRC depending on *RAS* mutation status.

Chemotherapy Responders had a significantly higher prevalence of *Ruminococcus* and *Lactobacillus*, whereas Non-responders had a higher prevalence of *Anaerococcus*, *Christensenellaceae*, *DTU089*, and *Porphyromonas*. The association between chemotherapy response and *Ruminococcus*, *Lactobacillus*, and *Anaerococcus* has been reported previously (22). *Porphyromonas* can also promote colon cancer by activating MAPK/ERK, JNK kinase, and NF-kB signaling (23). Further investigations of *Christensenellaceae* and *DTU089* are warranted to elucidate their clinical implications.

Notably, this study revealed individual changes in *Bacteroides* and *Bifidobacterium*—which are representative and well-known gut microorganisms in CRC (1, 7, 11), depending on whether the disease was controlled. *Bifidobacterium*, a beneficial microorganism, significantly decreased, and *Bacteroides*, a harmful microorganism, numerically increased during disease progression. Dynamic changes in the gut microbiome, as well as the baseline gut microbiome, may reflect cancer status.

Many studies investigating the interaction between the gut microbiome and cancer therapy have focused primarily on immune checkpoint inhibitors (24, 25). Several studies, including those reporting oxaliplatin-induced changes in gut microbiota and immune markers in the murine colon, as well as investigations of systemic immune responses following oxaliplatin-based neoadjuvant therapy, have suggested potential links between conventional chemotherapy, the gut microbiome, and host immunity (26, 27). However, the mechanistic interplay between conventional chemotherapeutic agents and the gut microbiota—particularly in modulating immune responses—has not been fully elucidated.

Our study offers preliminary observations that may contribute to a broader understanding of this area by exploring potential associations between gut microbial profiles and chemotherapy response in patients with CRC. Further research will be necessary to clarify whether chemotherapy-related immune modulation is influenced by the gut microbiota, which could potentially reveal novel aspects of treatment response or resistance.

This study had several limitations that should be acknowledged when interpreting the findings. First, the relatively small sample size constrained the statistical power of the analyses and limited our ability to conduct a comprehensive and robust assessment of changes in gut microbiome diversity across clinical subgroups. As a result, the observed associations should be considered exploratory and hypothesis-generating rather than conclusive. Further studies involving larger and independent cohorts are needed to validate these preliminary findings and better understand the potential clinical relevance of the microbiome signatures identified in this study.

Second, this study was conducted in a Korean population of East Asian descent, which may limit the generalizability of the findings to other ethnic groups. Moreover, data on key confounding variables—including socioeconomic status, marital status, use of medications or dietary supplements, and detailed lifestyle or dietary patterns—were not collected, which may have influenced gut microbiome composition.

Third, the timing of follow-up sample collection was not standardized across all patients due to practical and clinical considerations, potentially leading to inconsistencies in temporal microbiome data and limiting longitudinal interpretations.

Lastly, while our study observed potential associations between specific gut microbial species and clinical features, including RAS mutation status, we did not perform mechanistic experiments to investigate how these microbes might influence RAS-related pathways in gastrointestinal cancers. As a pilot exploratory study, our findings are preliminary and should be interpreted with caution. Further functional studies are warranted to elucidate the underlying biological mechanisms and to determine whether these microbes play a causal role in modulating RAS signaling or cancer progression. Future investigations should include shotgun metagenomics, metabolomics, and functional validation to clarify their relevance in CRC outcomes.

In conclusion, this pilot study sheds light on the intricate prognostic value of the gut microbiome and its association with *RAS* mutations in patients with mCRC treated with first-line chemotherapy. These findings provide a foundation for future studies of the gut microbiome.

## Data Availability

The datasets presented in this study can be found in online repositories. The names of the repository/repositories and accession number(s) can be found below: https://www.ncbi.nlm.nih.gov/, PRJNA1214585.
