# Supplementary material for: Gut microbiome with RAS mutation and chemotherapy response in patients with advanced or metastatic colorectal cancer: a pilot, exploratory study
Source: Front Oncol. 2025 Sep 1;15:1565661. doi: 10.3389/fonc.2025.1565661 (PMC12433852; doi:10.3389/fonc.2025.1565661)
Supplement: Supplementary file 1 [file DataSheet1.pdf]

Supplementary Material

Supplementary figure 1.

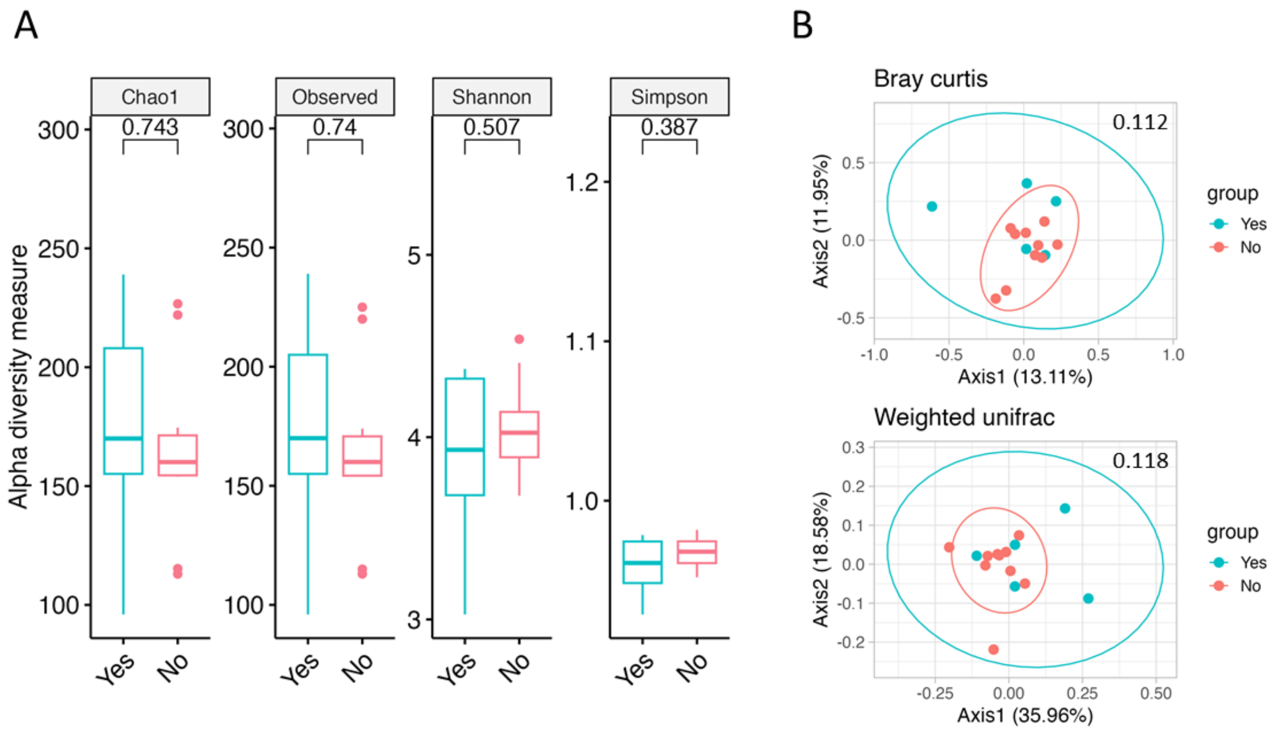

Supplementary Figure 1. The relationship between gut microbiome diversity and alcohol consumption (A) Alpha diversity metrics—Chao1, observed ASVs, Shannon, and Simpson indices—are compared between drinkers (green) and non-drinkers (red). (B) Beta diversity is visualized using PCoA plots for Bray–Curtis (top) and weighted UniFrac (bottom) distances. Each point represents a sample color-coded as a drinker (green) or nondrinker (red).

Supplementary figure 2.

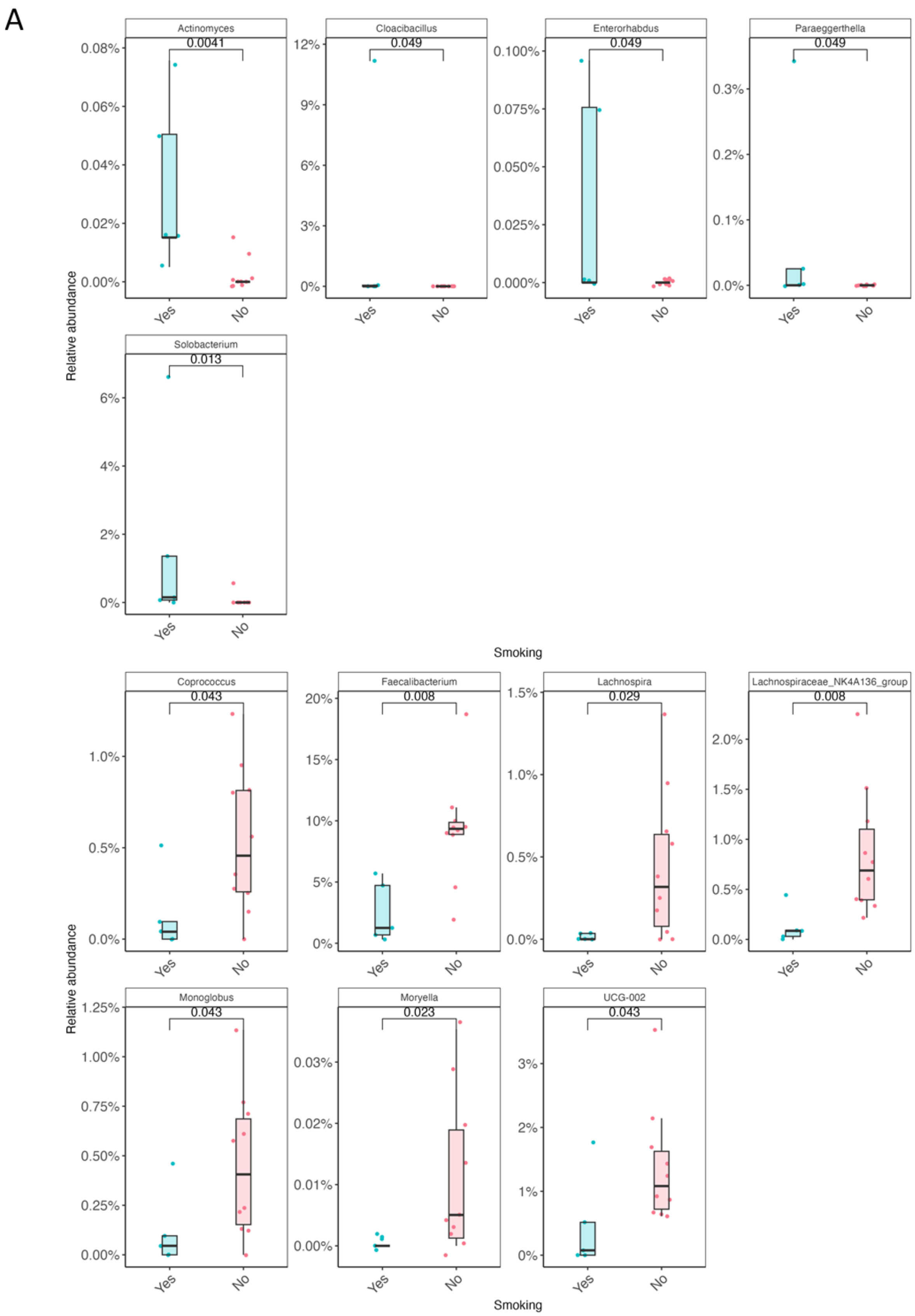

This is a provisional file, not the final typeset article

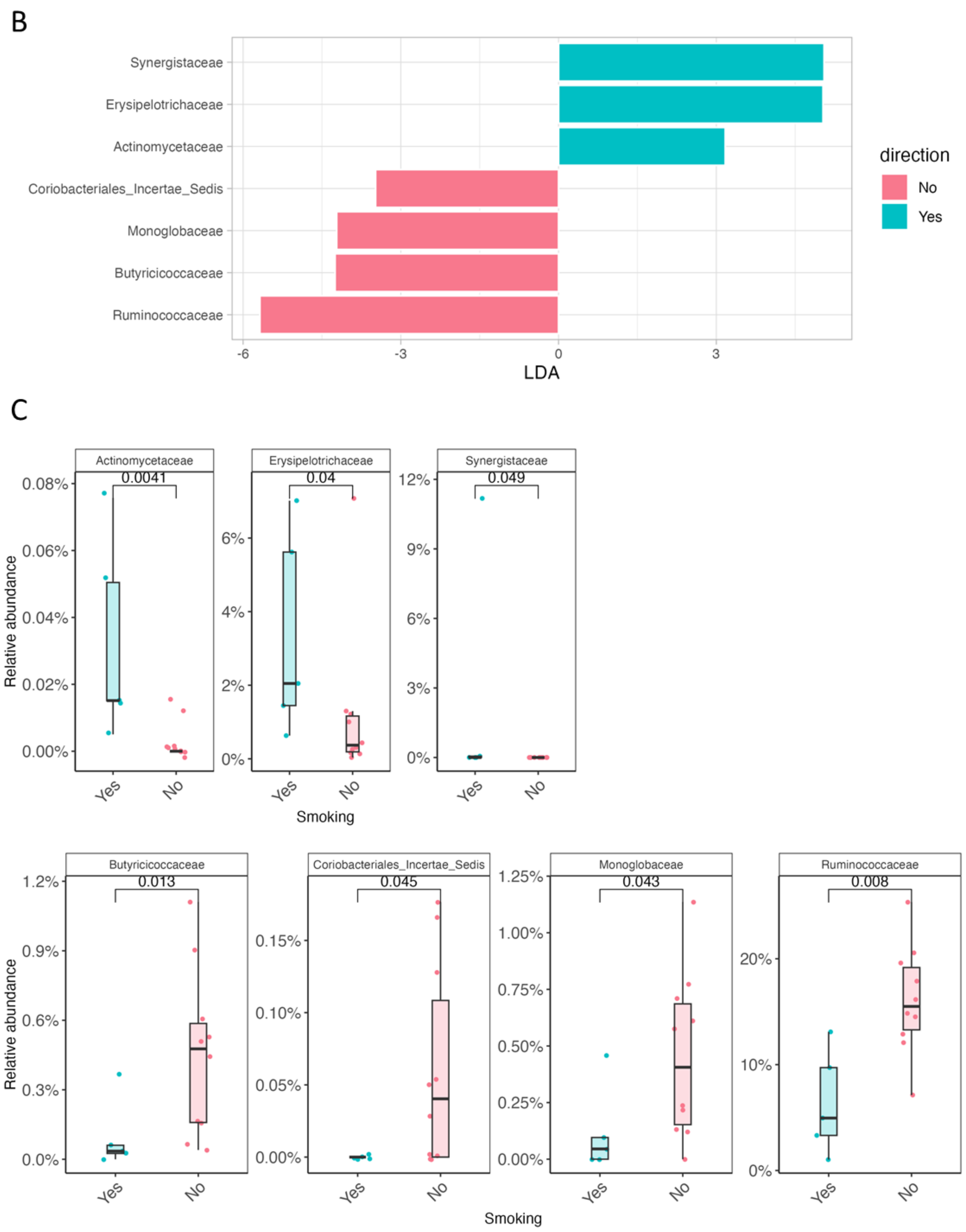

Supplementary Figure 2. The gut microbiome variations in patients with colorectal cancer based on smoking status. (A) At the genus level, distinct taxa are differentially abundant between smokers

(green) and never-smokers (red), (B) Family-level LEfSe analysis highlights specific taxa with varying abundances in smokers (green) versus never-smokers (red), (C) Identified taxa at the family level show differential presence in smokers (green) and never-smokers (red).

This is a provisional file, not the final typeset article

Supplementary Figure 3.

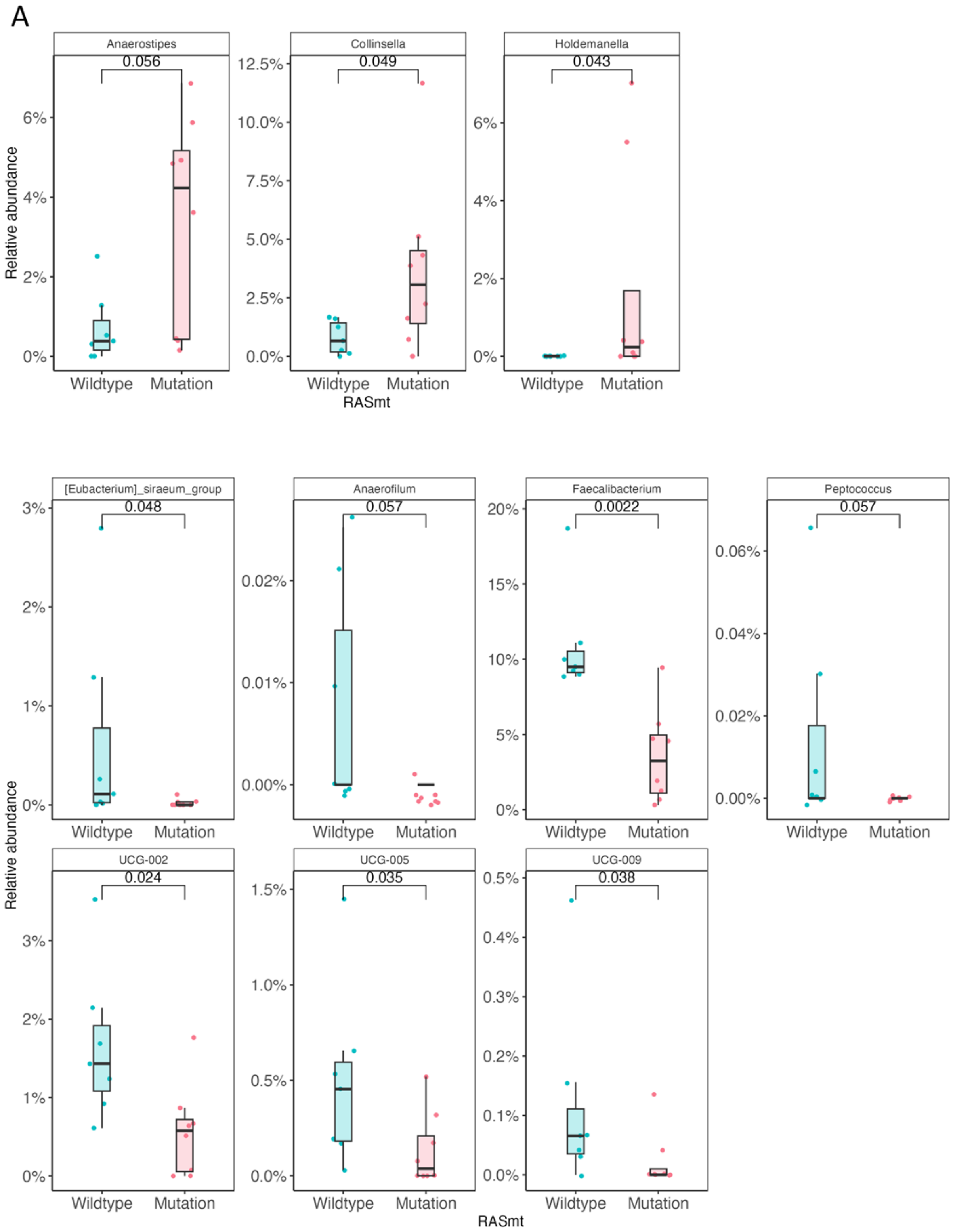

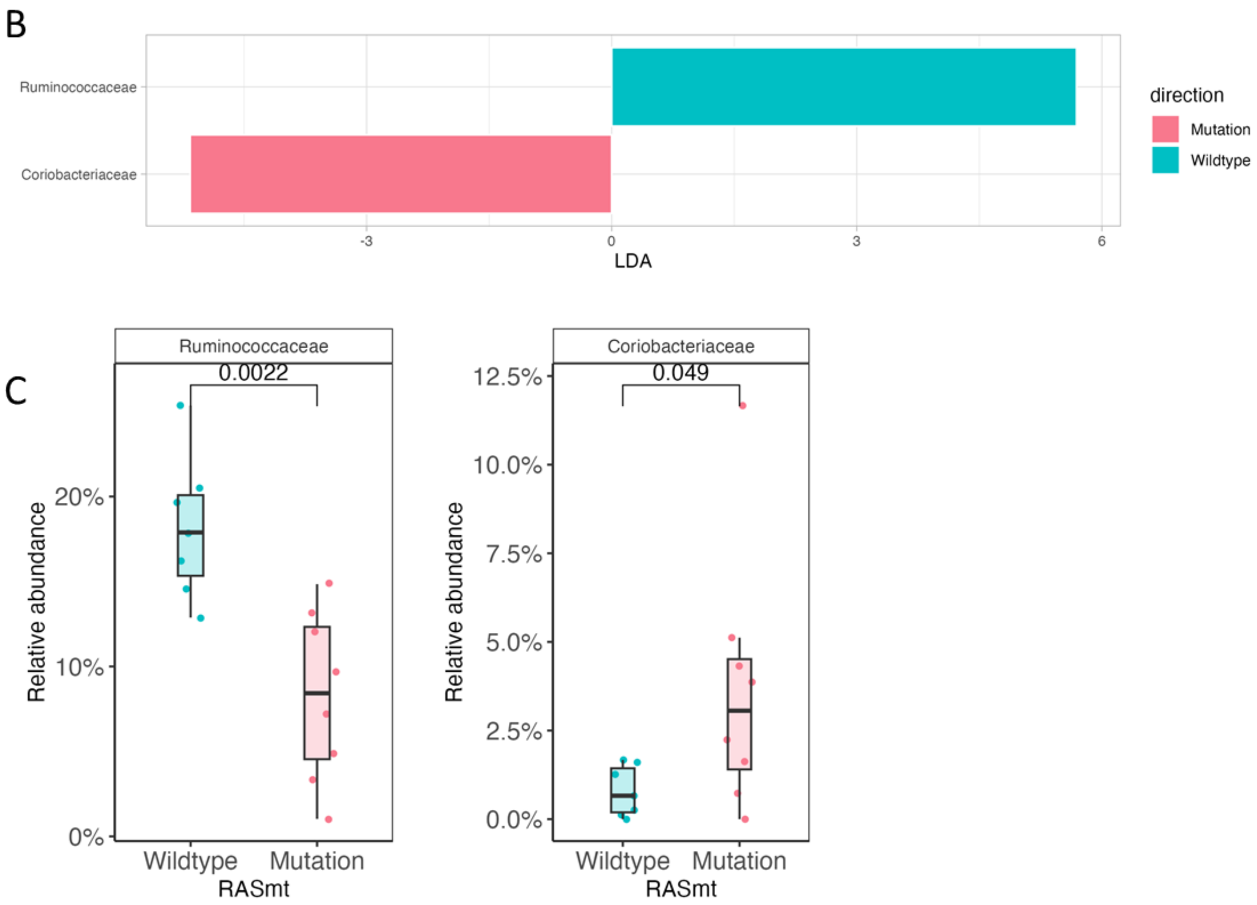

Supplementary Figure 3. Gut microbiome variations in patients with colorectal cancer based on the *RAS* mutation status. (A) At the genus level, distinct taxa were differentially abundant between *RAS* wild-type (green) and *RAS*-mutated (red) colorectal cancer cases. (B) Family level LEfSe analysis identified taxa with differential abundance in *RAS* wild-type (green) and *RAS*-mutated (red) colorectal cancer. (C) Family level identified taxa with differential abundance in *RAS* wild-type (green) and *RAS*-mutated (red) CRC.

This is a provisional file, not the final typeset article

Supplementary Figure 4.

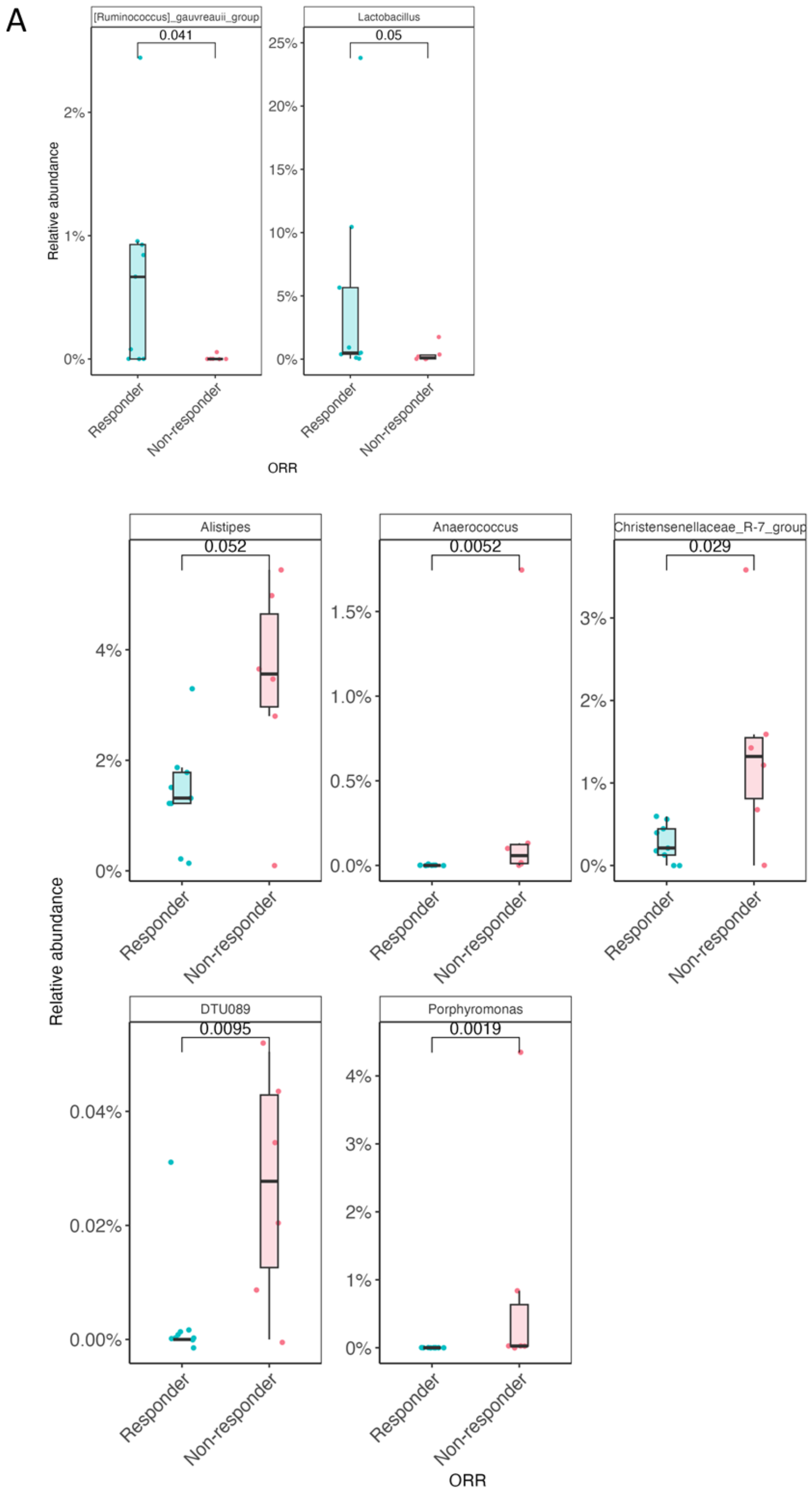

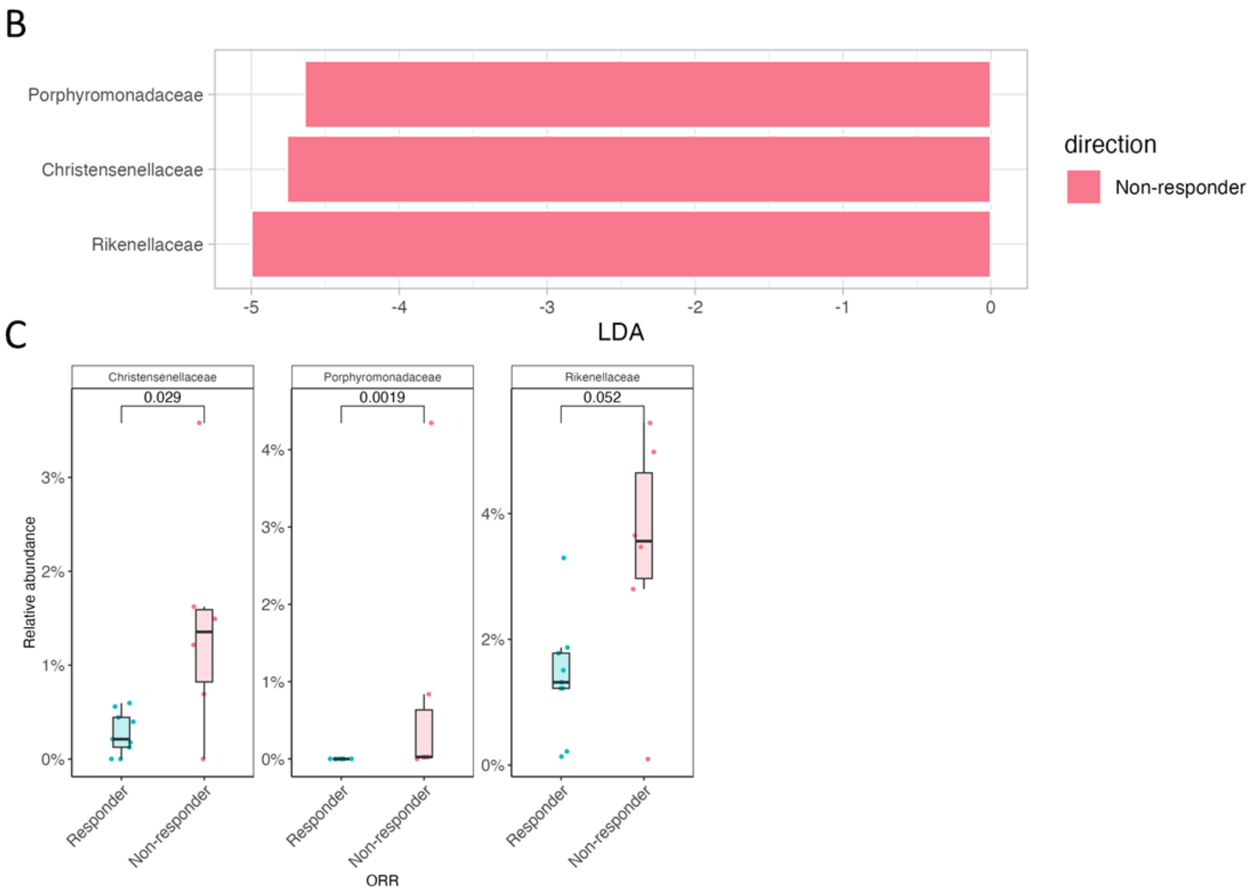

Supplementary Figure 4. The variations in the gut microbiome of patients with colorectal cancer based on their response to chemotherapy. (A) At the genus level, distinct taxa are differentially abundant between responders (green) and non-responders (red), (B) Family-level LEfSe analysis highlights specific taxa with varying abundances in non-responders (red). Data for the respondents were not identified. (C) At the family level, distinct taxa displayed differential presence between responders (green) and non-responders (red).

This is a provisional file, not the final typeset article

Supplementary figure 5.

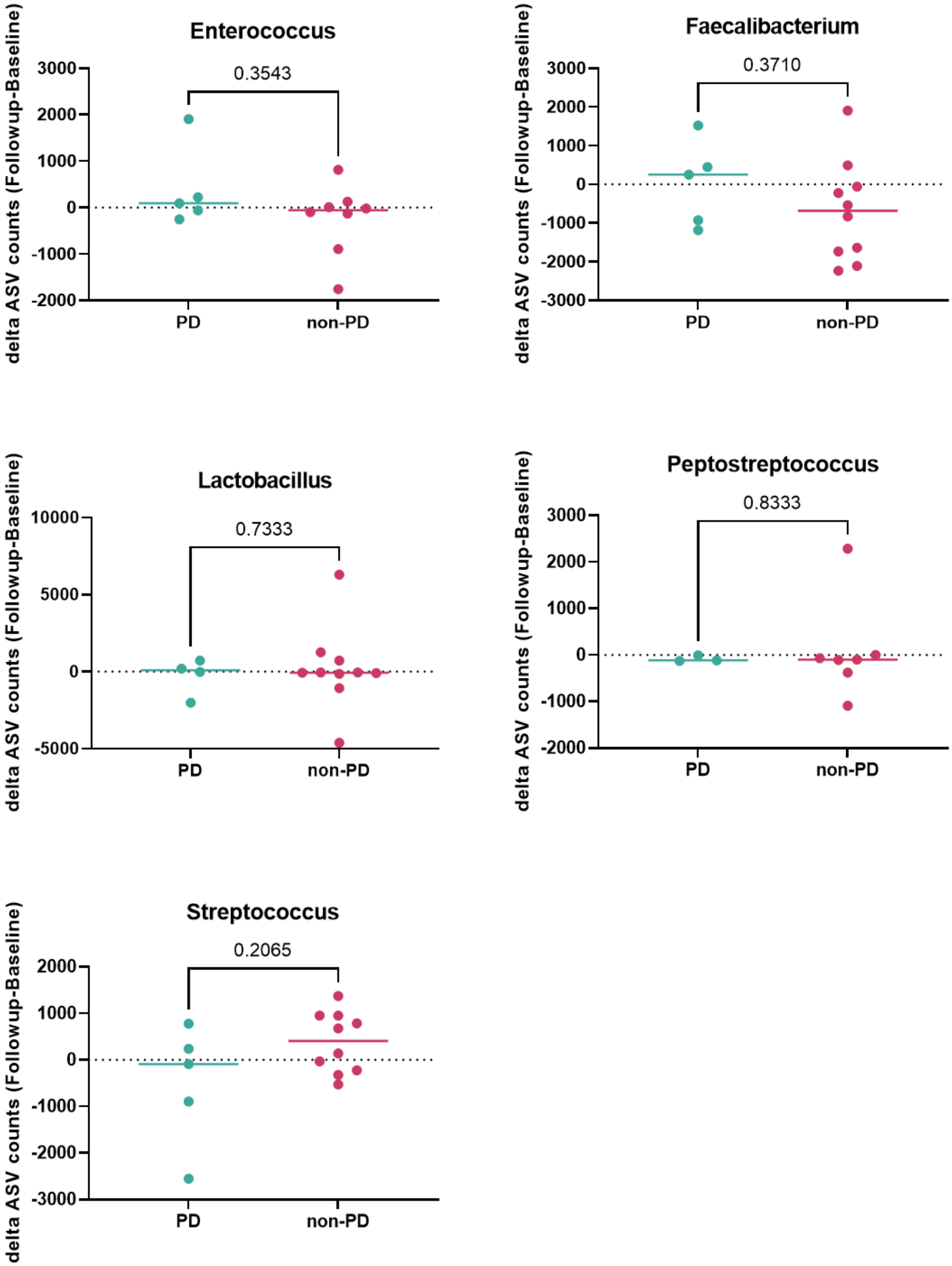

Supplementary figure 5.

Supplementary Figure 5. Gut microbiome analysis according to disease control during the follow-up. Relative abundances of *Enterococcus*, *Faecalibacterium*, *Lactobacillus*, *Peptostreptococcus*, and *Streptococcus* were compared between patients with progressive disease (PD) (green) and those without disease progression (non-PD) (red).

This is a provisional file, not the final typeset article
